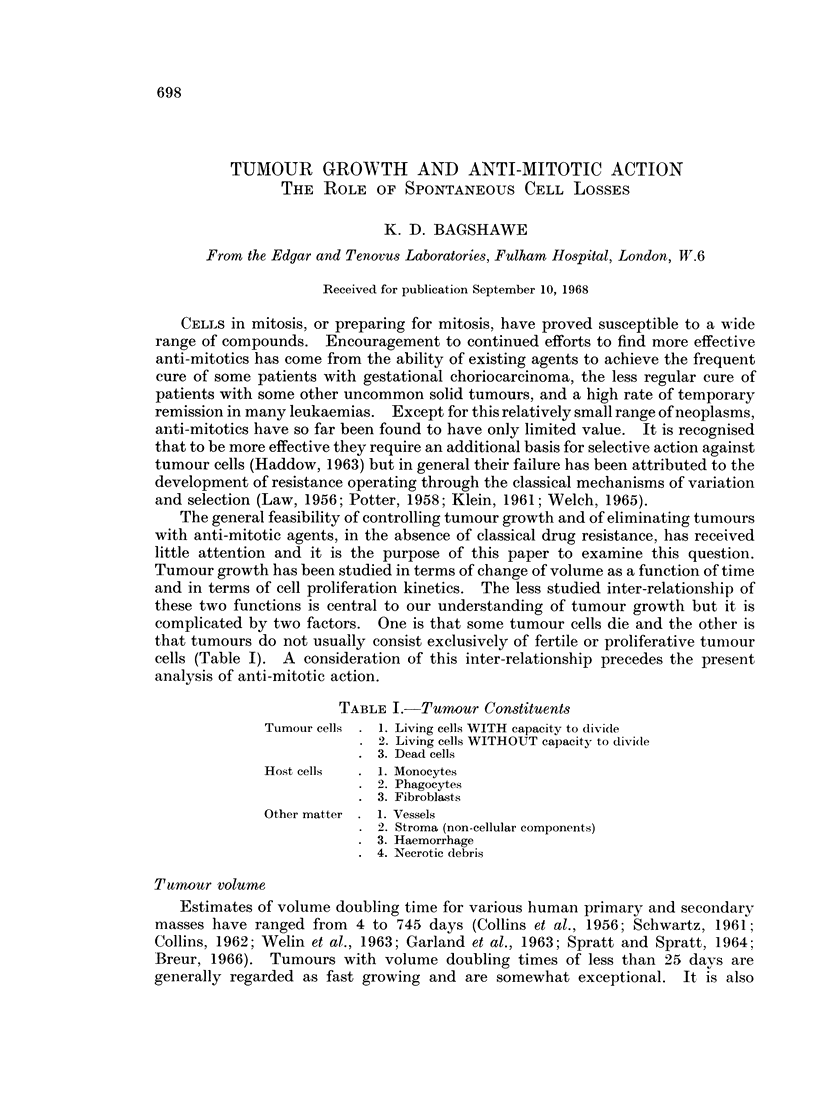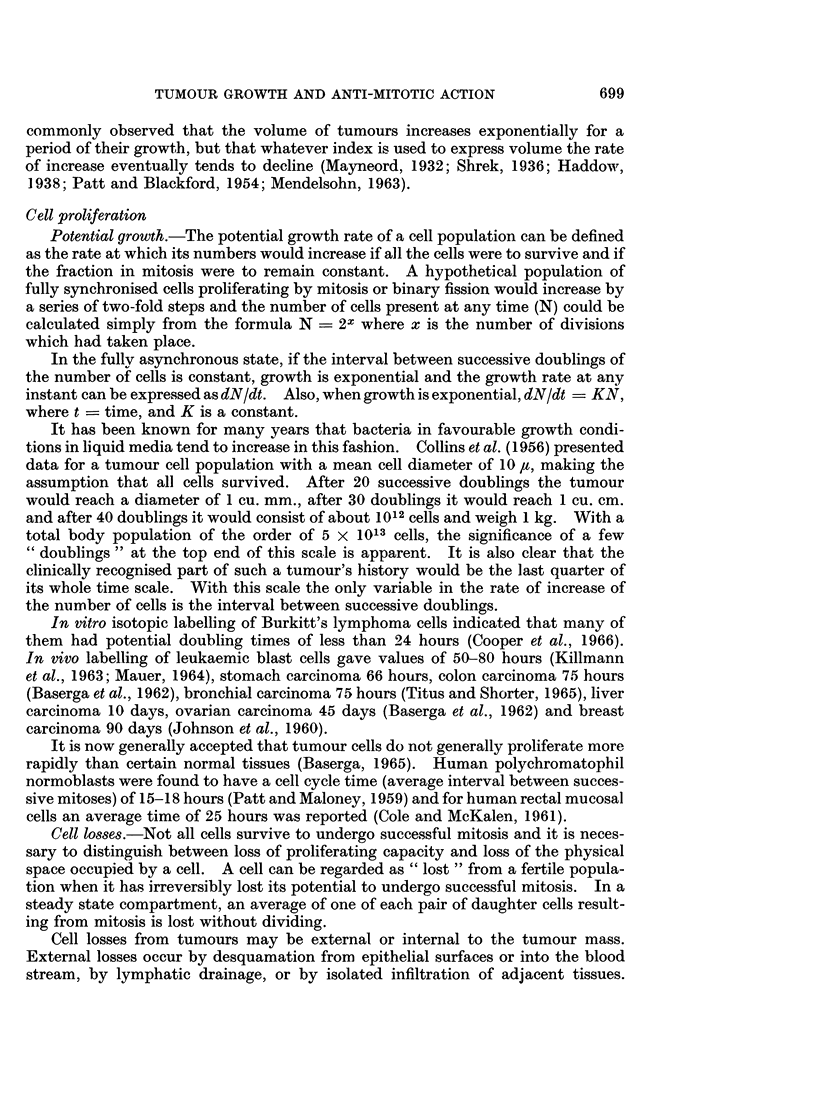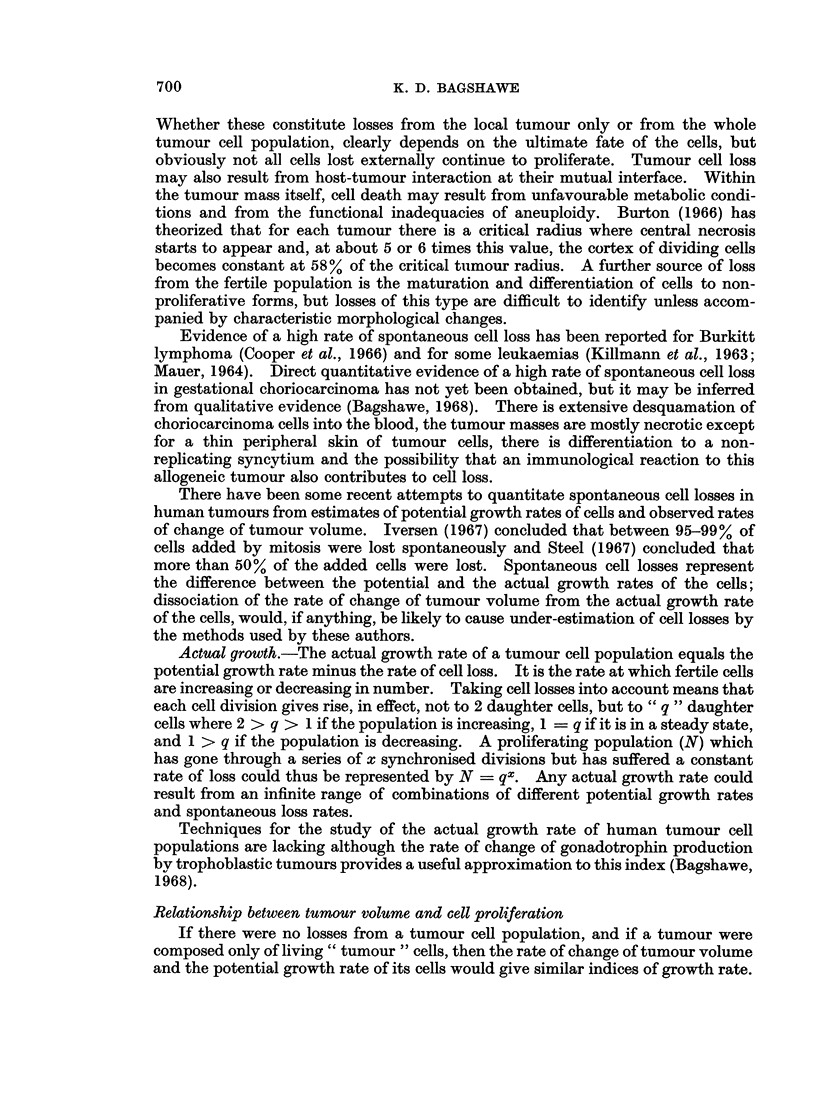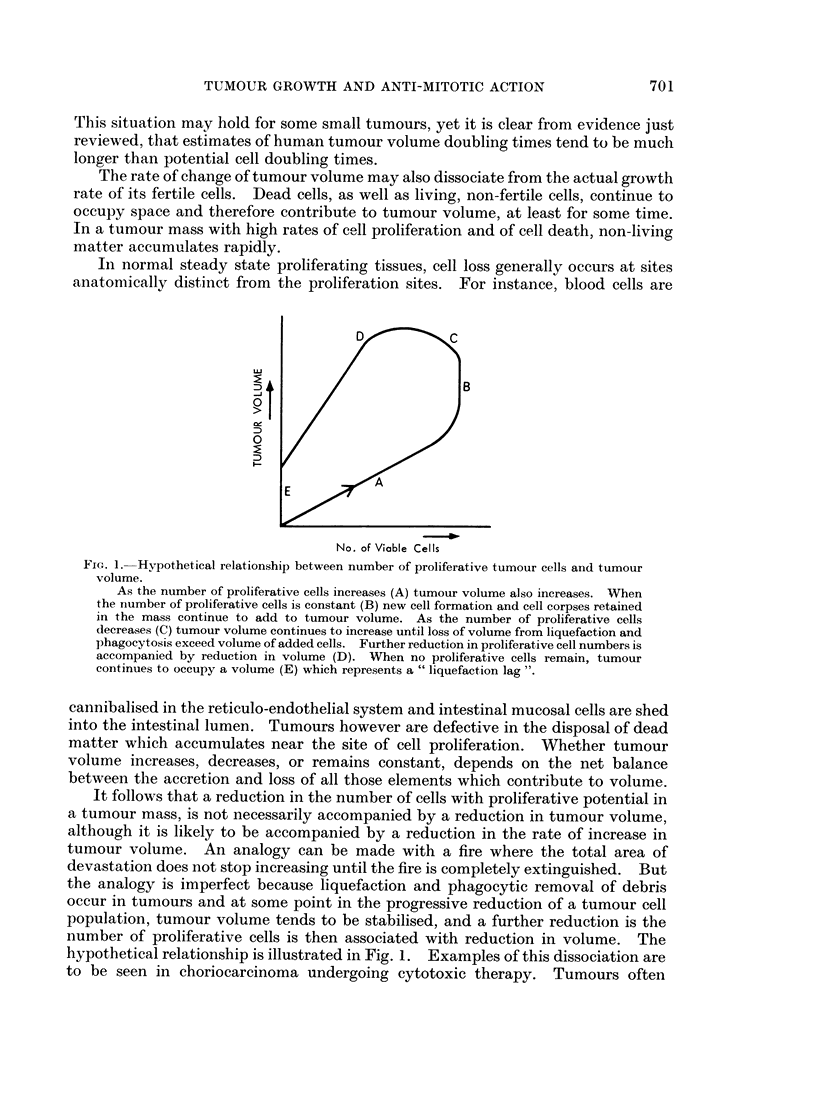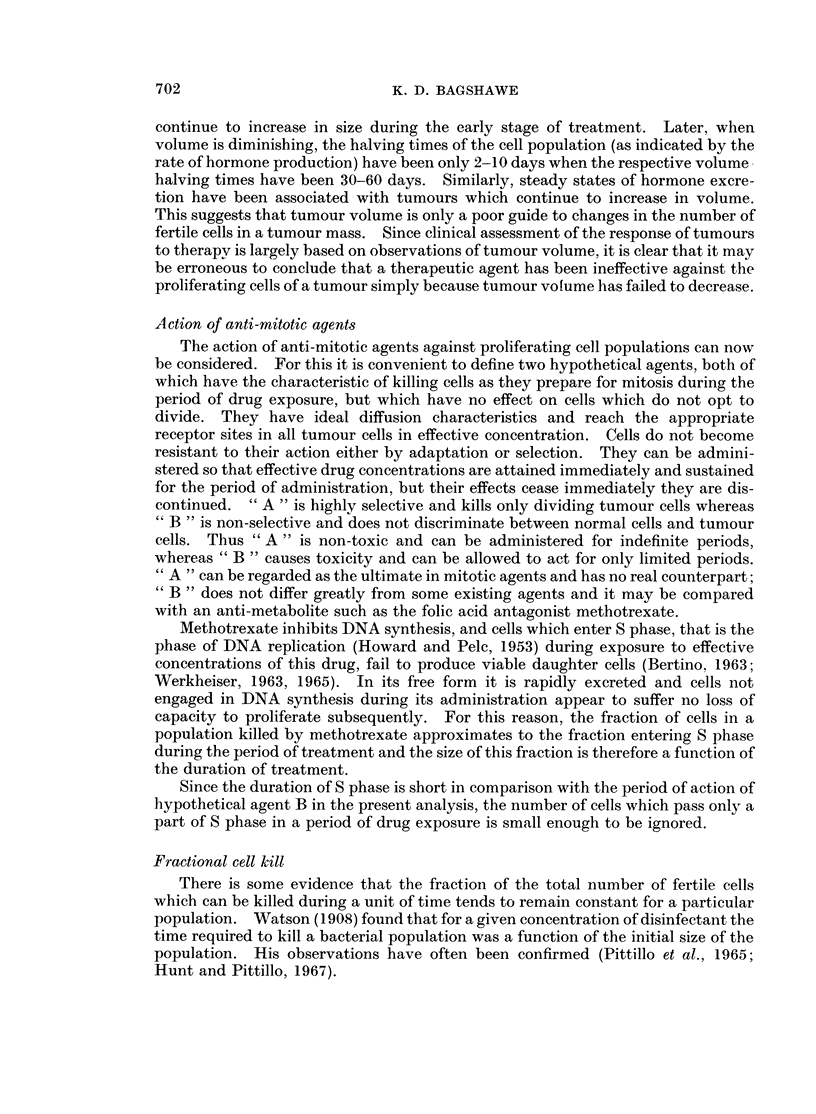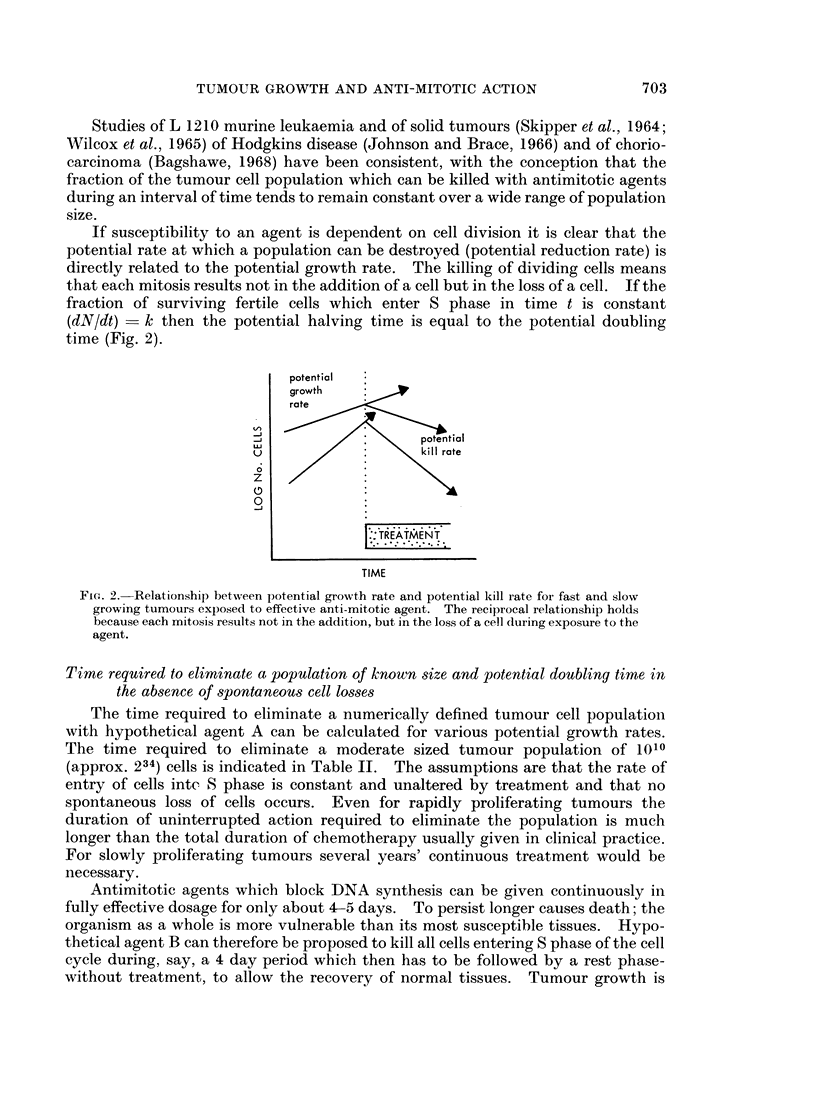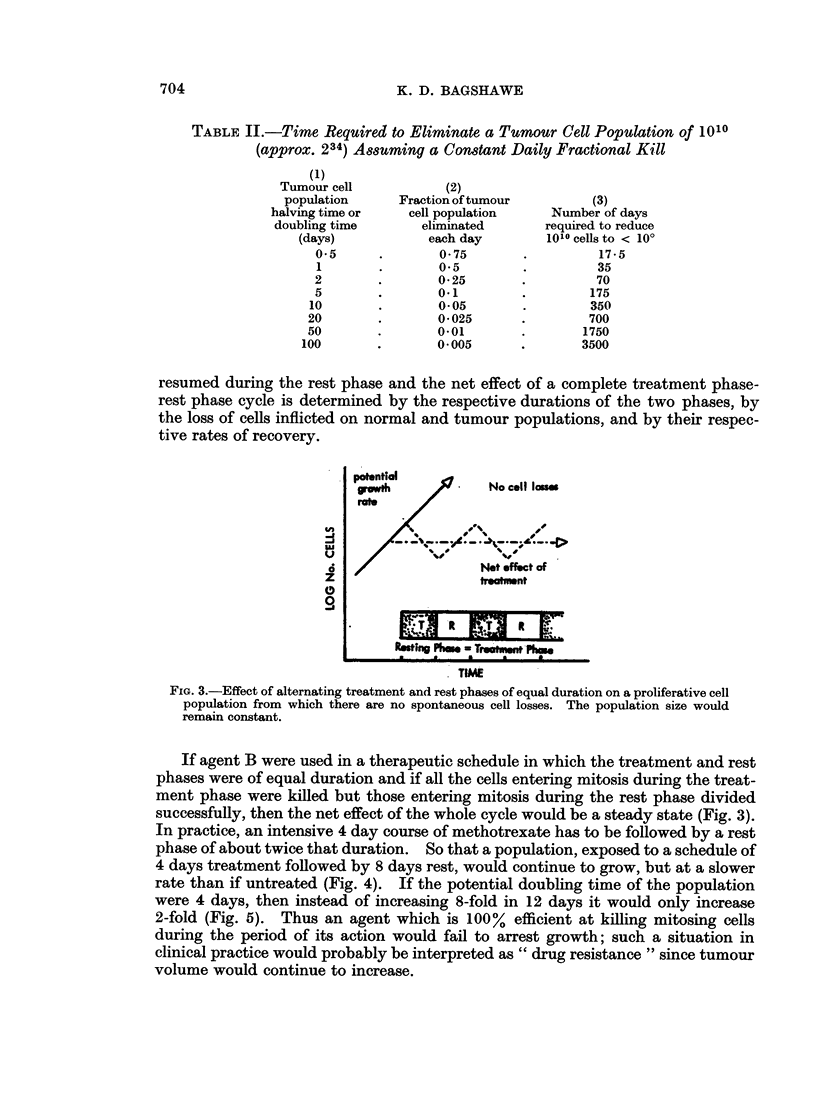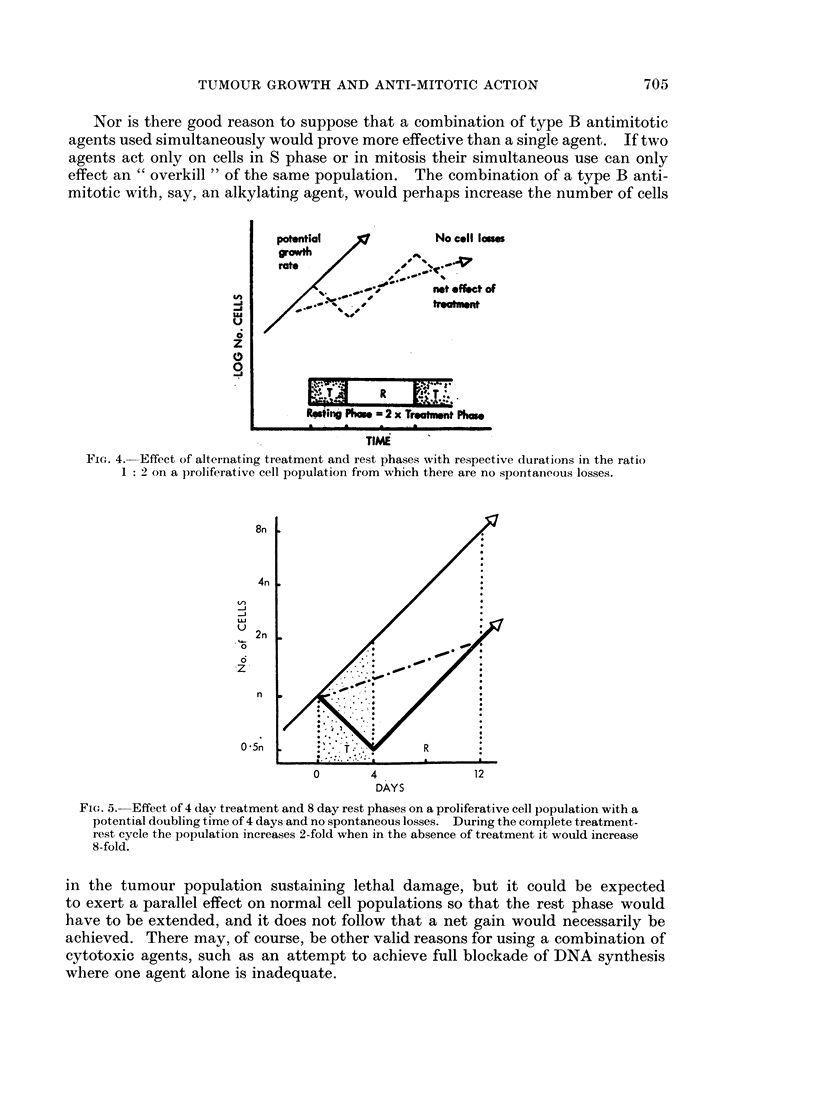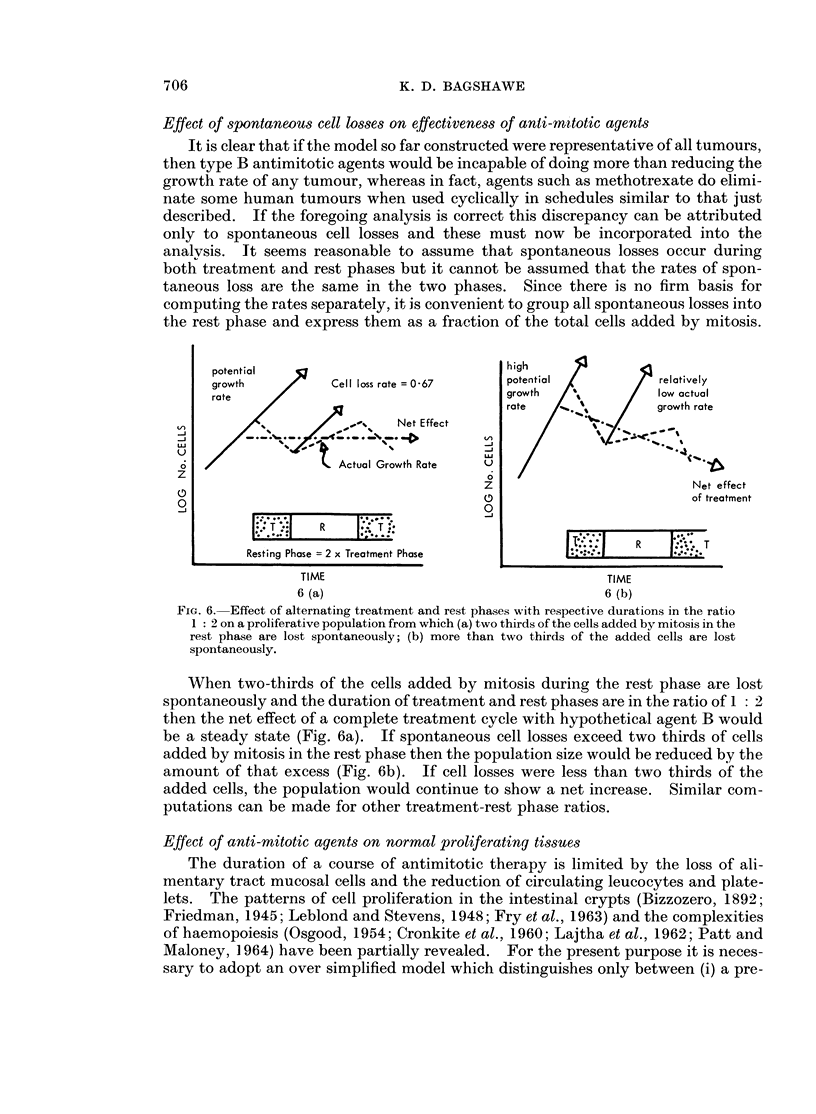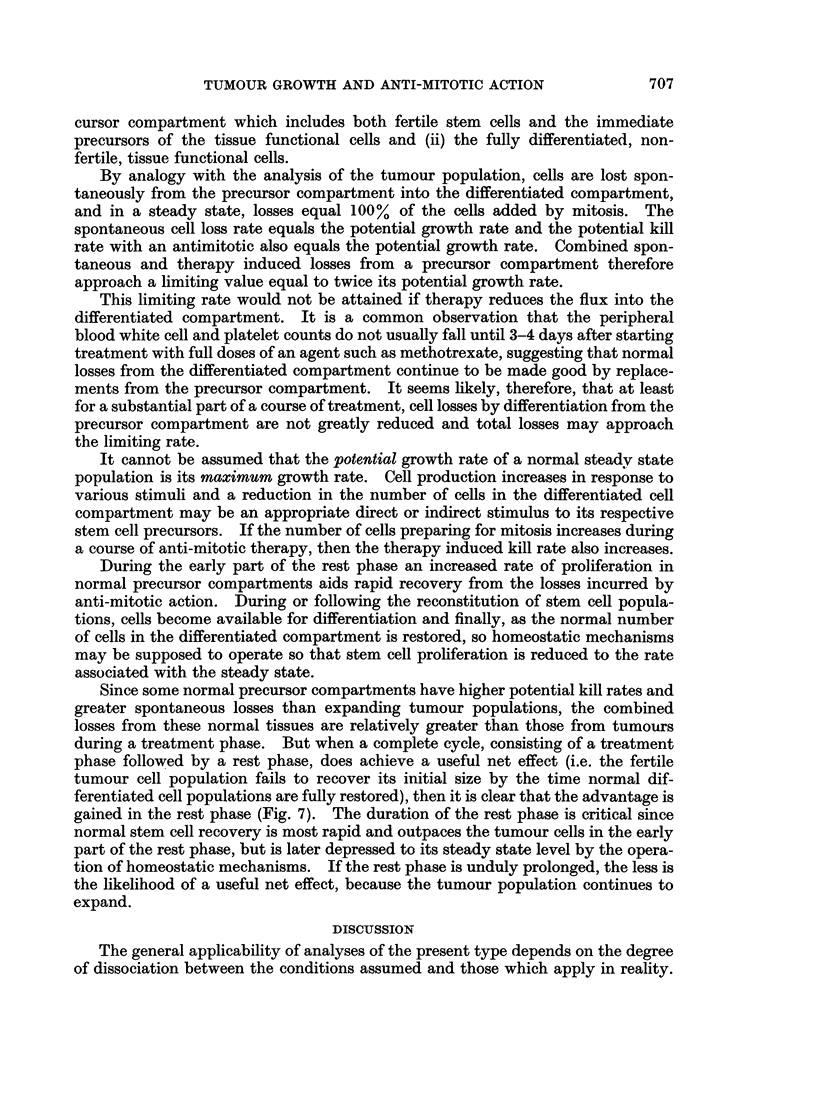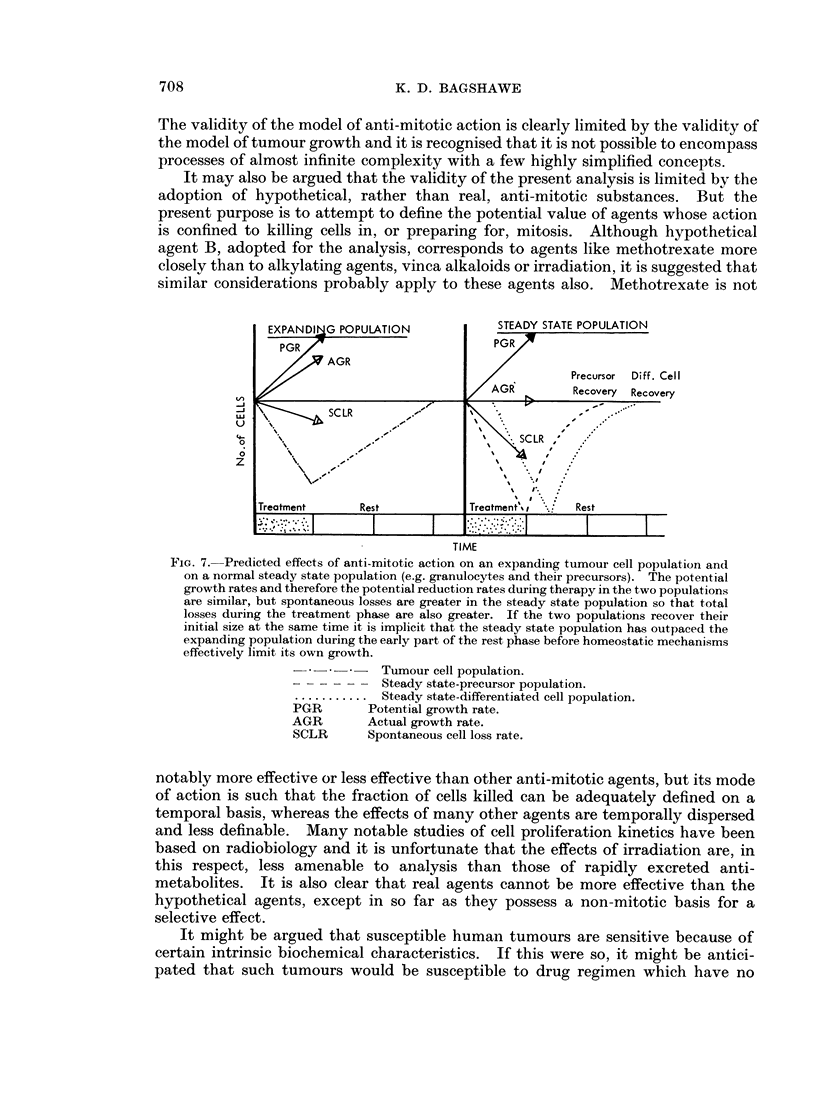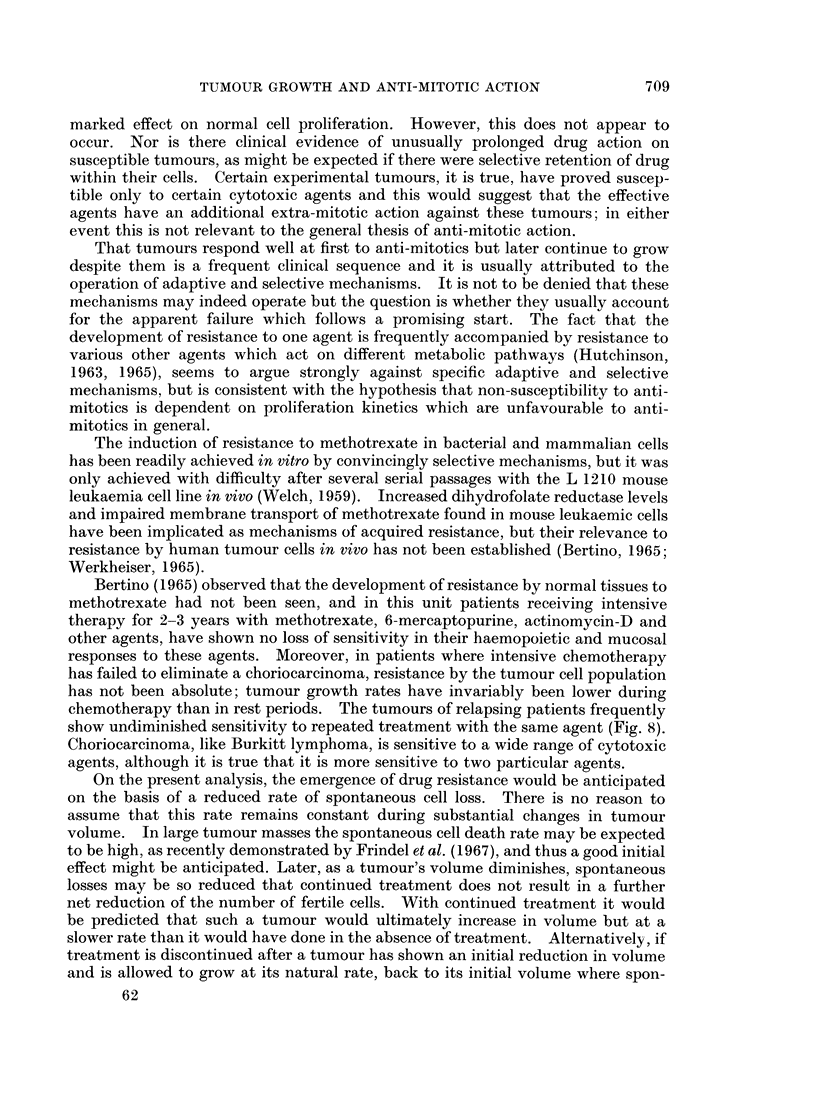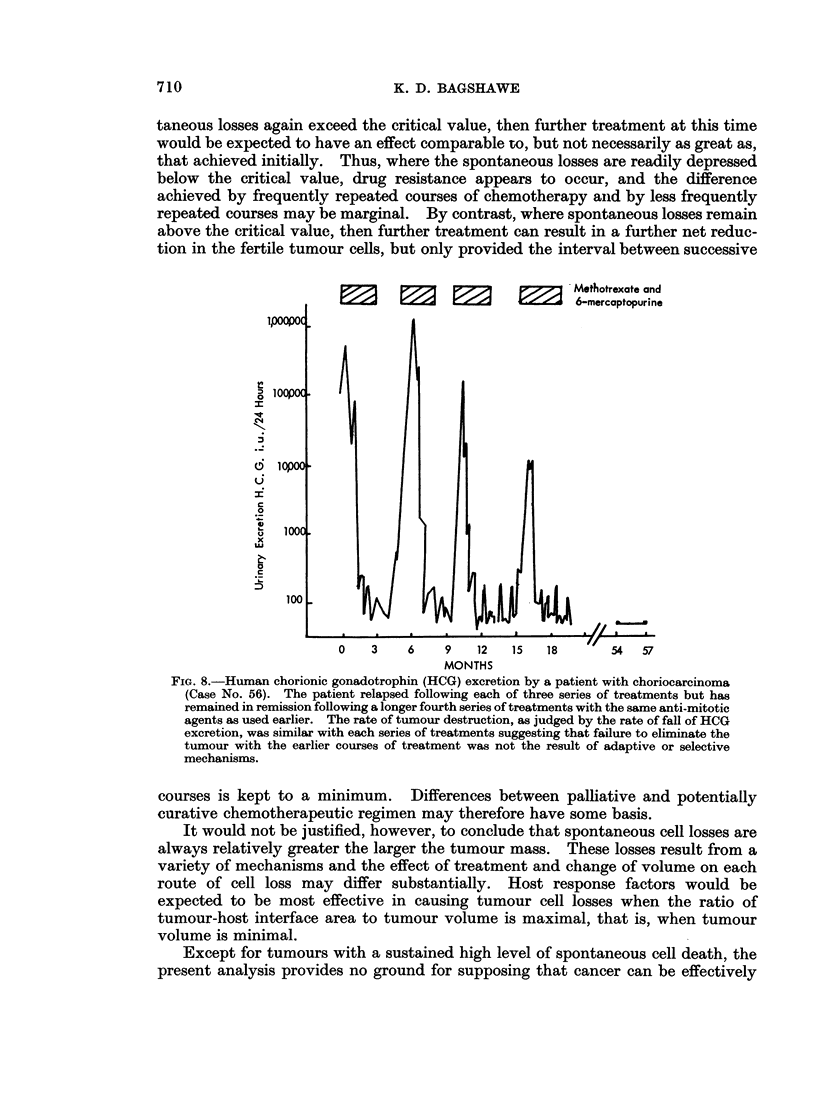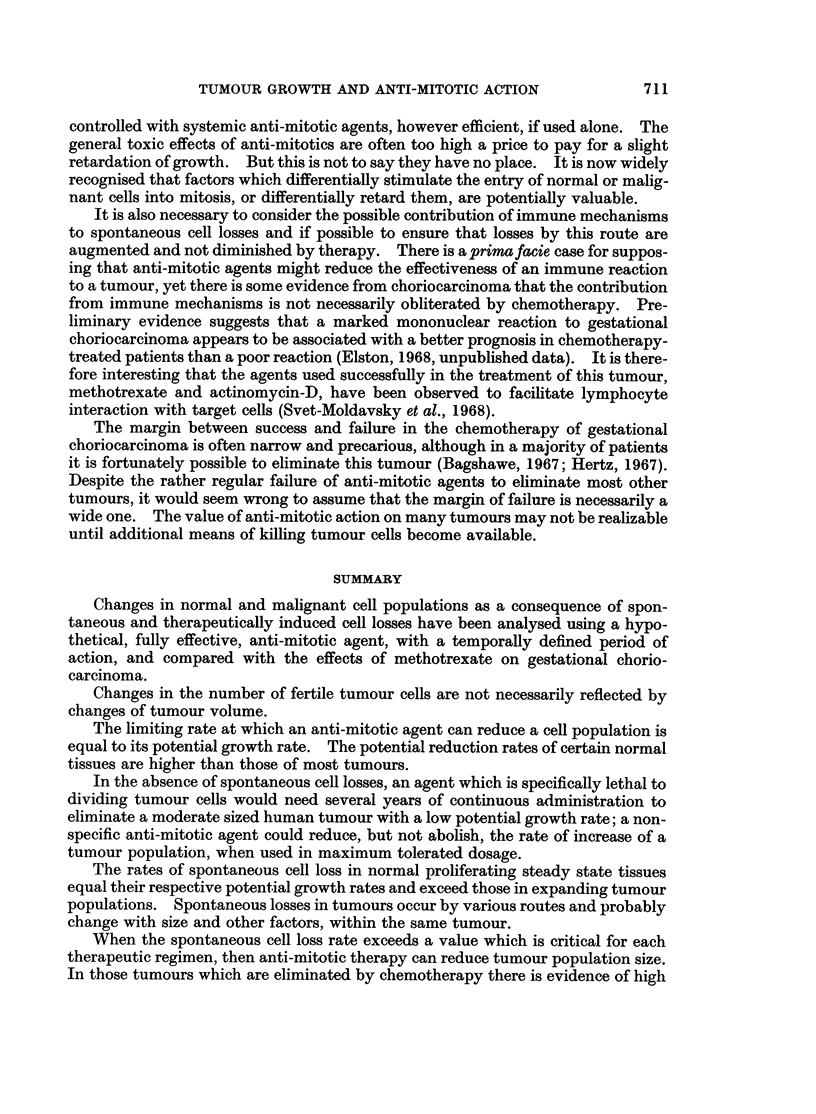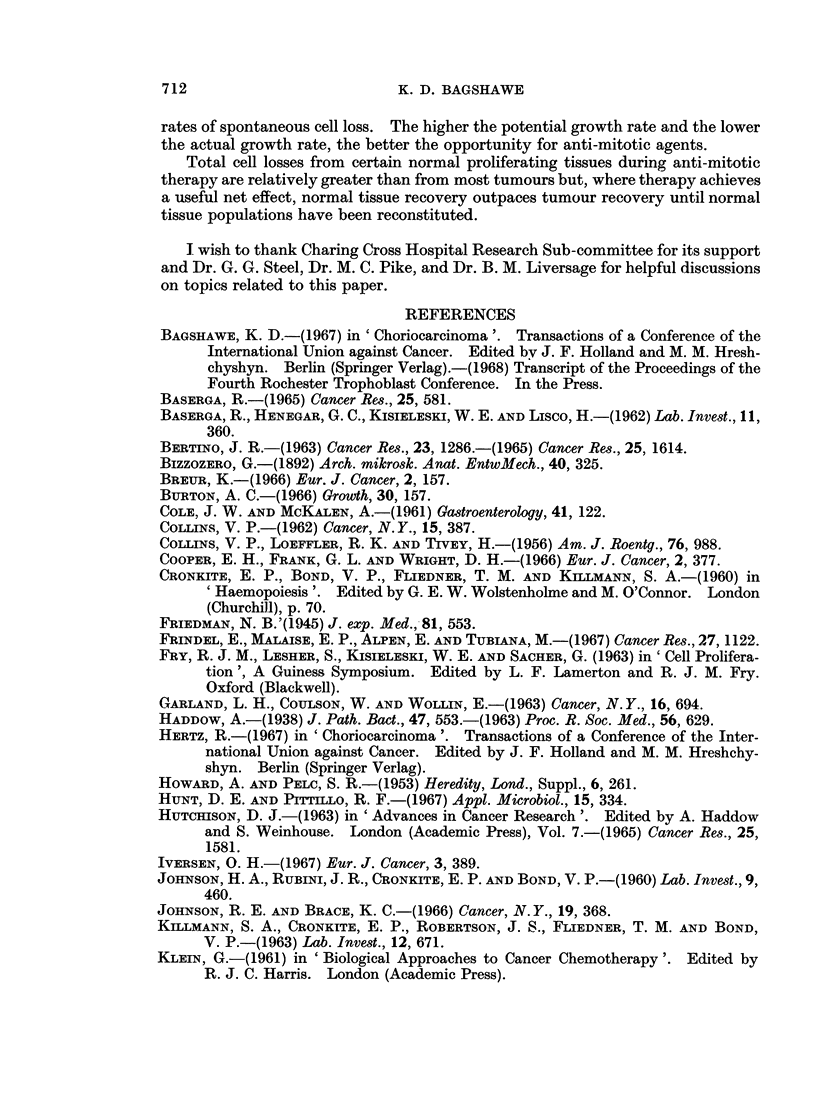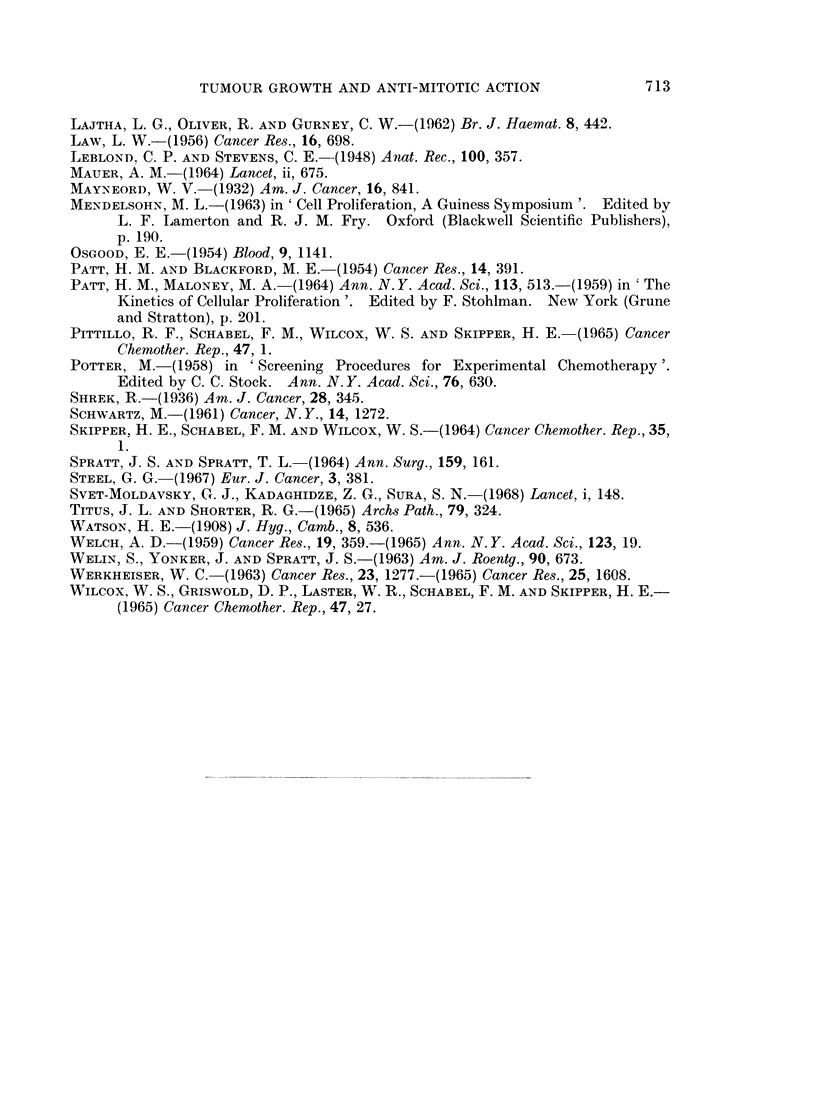# Tumour growth and anti-mitotic action. The role of spontaneous cell losses.

**DOI:** 10.1038/bjc.1968.83

**Published:** 1968-12

**Authors:** K. D. Bagshawe


### TUMOUR GROWTH AND ANTI-MITOTIC ACTION
### THE ROLE OF SPONTANEOUS CELL LOSSES

K. D. BAGSHAWE

*From the Edgar and Tenovus Laboratories, Fulham Hospital, London, W.6*

Received for publication September 10, 1968

CELLS in mitosis, or preparing for mitosis, have proved susceptible to a wide range of compounds. Encouragement to continued efforts to find more effective anti-mitotics has come from the ability of existing agents to achieve the frequent cure of some patients with gestational choriocarcinoma, the less regular cure of patients with some other uncommon solid tumours, and a high rate of temporary remission in many leukaemias. Except for this relatively small range of neoplasms, anti-mitotics have so far been found to have only limited value. It is recognised that to be more effective they require an additional basis for selective action against tumour cells (Haddow, 1963) but in general their failure has been attributed to the development of resistance operating through the classical mechanisms of variation and selection (Law, 1956; Potter, 1958; Klein, 1961; Welch, 1965).

The general feasibility of controlling tumour growth and of eliminating tumours with anti-mitotic agents, in the absence of classical drug resistance, has received little attention and it is the purpose of this paper to examine this question. Tumour growth has been studied in terms of change of volume as a function of time and in terms of cell proliferation kinetics. The less studied inter-relationship of these two functions is central to our understanding of tumour growth but it is complicated by two factors. One is that some tumour cells die and the other is that tumours do not usually consist exclusively of fertile or proliferative tumour cells (Table I). A consideration of this inter-relationship precedes the present analysis of anti-mitotic action.

TABLE I.—*Tumour Constituents*

| | |
|---|---|
| Tumour cells | 1. Living cells WITH capacity to divide |
| | 2. Living cells WITHOUT capacity to divide |
| | 3. Dead cells |
| Host cells | 1. Monocytes |
| | 2. Phagocytes |
| | 3. Fibroblasts |
| Other matter | 1. Vessels |
| | 2. Stroma (non-cellular components) |
| | 3. Haemorrhage |
| | 4. Necrotic debris |

*Tumour volume*

Estimates of volume doubling time for various human primary and secondary masses have ranged from 4 to 745 days (Collins *et al.*, 1956; Schwartz, 1961; Collins, 1962; Welin *et al.*, 1963; Garland *et al.*, 1963; Spratt and Spratt, 1964; Breur, 1966). Tumours with volume doubling times of less than 25 days are generally regarded as fast growing and are somewhat exceptional. It is also commonly observed that the volume of tumours increases exponentially for a period of their growth, but that whatever index is used to express volume the rate of increase eventually tends to decline (Mayneord, 1932; Shrek, 1936; Haddow, 1938; Patt and Blackford, 1954; Mendelsohn, 1963).

*Cell proliferation*

*Potential growth.*—The potential growth rate of a cell population can be defined as the rate at which its numbers would increase if all the cells were to survive and if the fraction in mitosis were to remain constant. A hypothetical population of fully synchronised cells proliferating by mitosis or binary fission would increase by a series of two-fold steps and the number of cells present at any time (N) could be calculated simply from the formula $N = 2^x$ where $x$ is the number of divisions which had taken place.

In the fully asynchronous state, if the interval between successive doublings of the number of cells is constant, growth is exponential and the growth rate at any instant can be expressed as $dN/dt$. Also, when growth is exponential, $dN/dt = KN$, where $t$ = time, and $K$ is a constant.

It has been known for many years that bacteria in favourable growth conditions in liquid media tend to increase in this fashion. Collins *et al.* (1956) presented data for a tumour cell population with a mean cell diameter of 10 $\mu$, making the assumption that all cells survived. After 20 successive doublings the tumour would reach a diameter of 1 cu. mm., after 30 doublings it would reach 1 cu. cm. and after 40 doublings it would consist of about $10^{12}$ cells and weigh 1 kg. With a total body population of the order of $5 \times 10^{13}$ cells, the significance of a few " doublings " at the top end of this scale is apparent. It is also clear that the clinically recognised part of such a tumour's history would be the last quarter of its whole time scale. With this scale the only variable in the rate of increase of the number of cells is the interval between successive doublings.

*In vitro* isotopic labelling of Burkitt's lymphoma cells indicated that many of them had potential doubling times of less than 24 hours (Cooper *et al.*, 1966). *In vivo* labelling of leukaemic blast cells gave values of 50–80 hours (Killmann *et al.*, 1963; Mauer, 1964), stomach carcinoma 66 hours, colon carcinoma 75 hours (Baserga *et al.*, 1962), bronchial carcinoma 75 hours (Titus and Shorter, 1965), liver carcinoma 10 days, ovarian carcinoma 45 days (Baserga *et al.*, 1962) and breast carcinoma 90 days (Johnson *et al.*, 1960).

It is now generally accepted that tumour cells do not generally proliferate more rapidly than certain normal tissues (Baserga, 1965). Human polychromatophil normoblasts were found to have a cell cycle time (average interval between successive mitoses) of 15–18 hours (Patt and Maloney, 1959) and for human rectal mucosal cells an average time of 25 hours was reported (Cole and McKalen, 1961).

*Cell losses.*—Not all cells survive to undergo successful mitosis and it is necessary to distinguish between loss of proliferating capacity and loss of the physical space occupied by a cell. A cell can be regarded as " lost " from a fertile population when it has irreversibly lost its potential to undergo successful mitosis. In a steady state compartment, an average of one of each pair of daughter cells resulting from mitosis is lost without dividing.

Cell losses from tumours may be external or internal to the tumour mass. External losses occur by desquamation from epithelial surfaces or into the blood stream, by lymphatic drainage, or by isolated infiltration of adjacent tissues.

Whether these constitute losses from the local tumour only or from the whole tumour cell population, clearly depends on the ultimate fate of the cells, but obviously not all cells lost externally continue to proliferate. Tumour cell loss may also result from host-tumour interaction at their mutual interface. Within the tumour mass itself, cell death may result from unfavourable metabolic conditions and from the functional inadequacies of aneuploidy. Burton (1966) has theorized that for each tumour there is a critical radius where central necrosis starts to appear and, at about 5 or 6 times this value, the cortex of dividing cells becomes constant at 58% of the critical tumour radius. A further source of loss from the fertile population is the maturation and differentiation of cells to non-proliferative forms, but losses of this type are difficult to identify unless accompanied by characteristic morphological changes.

Evidence of a high rate of spontaneous cell loss has been reported for Burkitt lymphoma (Cooper *et al.*, 1966) and for some leukaemias (Killmann *et al.*, 1963; Mauer, 1964). Direct quantitative evidence of a high rate of spontaneous cell loss in gestational choriocarcinoma has not yet been obtained, but it may be inferred from qualitative evidence (Bagshawe, 1968). There is extensive desquamation of choriocarcinoma cells into the blood, the tumour masses are mostly necrotic except for a thin peripheral skin of tumour cells, there is differentiation to a non-replicating syncytium and the possibility that an immunological reaction to this allogeneic tumour also contributes to cell loss.

There have been some recent attempts to quantitate spontaneous cell losses in human tumours from estimates of potential growth rates of cells and observed rates of change of tumour volume. Iversen (1967) concluded that between 95–99% of cells added by mitosis were lost spontaneously and Steel (1967) concluded that more than 50% of the added cells were lost. Spontaneous cell losses represent the difference between the potential and the actual growth rates of the cells; dissociation of the rate of change of tumour volume from the actual growth rate of the cells, would, if anything, be likely to cause under-estimation of cell losses by the methods used by these authors.

*Actual growth.*—The actual growth rate of a tumour cell population equals the potential growth rate minus the rate of cell loss. It is the rate at which fertile cells are increasing or decreasing in number. Taking cell losses into account means that each cell division gives rise, in effect, not to 2 daughter cells, but to " $q$ " daughter cells where $2 > q > 1$ if the population is increasing, $1 = q$ if it is in a steady state, and $1 > q$ if the population is decreasing. A proliferating population ($N$) which has gone through a series of $x$ synchronised divisions but has suffered a constant rate of loss could thus be represented by $N = q^x$. Any actual growth rate could result from an infinite range of combinations of different potential growth rates and spontaneous loss rates.

Techniques for the study of the actual growth rate of human tumour cell populations are lacking although the rate of change of gonadotrophin production by trophoblastic tumours provides a useful approximation to this index (Bagshawe, 1968).

*Relationship between tumour volume and cell proliferation*

If there were no losses from a tumour cell population, and if a tumour were composed only of living " tumour " cells, then the rate of change of tumour volume and the potential growth rate of its cells would give similar indices of growth rate.

This situation may hold for some small tumours, yet it is clear from evidence just reviewed, that estimates of human tumour volume doubling times tend to be much longer than potential cell doubling times.

The rate of change of tumour volume may also dissociate from the actual growth rate of its fertile cells. Dead cells, as well as living, non-fertile cells, continue to occupy space and therefore contribute to tumour volume, at least for some time. In a tumour mass with high rates of cell proliferation and of cell death, non-living matter accumulates rapidly.

In normal steady state proliferating tissues, cell loss generally occurs at sites anatomically distinct from the proliferation sites. For instance, blood cells are

FIG. 1.—Hypothetical relationship between number of proliferative tumour cells and tumour volume.

As the number of proliferative cells increases (A) tumour volume also increases. When the number of proliferative cells is constant (B) new cell formation and cell corpses retained in the mass continue to add to tumour volume. As the number of proliferative cells decreases (C) tumour volume continues to increase until loss of volume from liquefaction and phagocytosis exceed volume of added cells. Further reduction in proliferative cell numbers is accompanied by reduction in volume (D). When no proliferative cells remain, tumour continues to occupy a volume (E) which represents a " liquefaction lag ".

cannibalised in the reticulo-endothelial system and intestinal mucosal cells are shed into the intestinal lumen. Tumours however are defective in the disposal of dead matter which accumulates near the site of cell proliferation. Whether tumour volume increases, decreases, or remains constant, depends on the net balance between the accretion and loss of all those elements which contribute to volume.

It follows that a reduction in the number of cells with proliferative potential in a tumour mass, is not necessarily accompanied by a reduction in tumour volume, although it is likely to be accompanied by a reduction in the rate of increase in tumour volume. An analogy can be made with a fire where the total area of devastation does not stop increasing until the fire is completely extinguished. But the analogy is imperfect because liquefaction and phagocytic removal of debris occur in tumours and at some point in the progressive reduction of a tumour cell population, tumour volume tends to be stabilised, and a further reduction is the number of proliferative cells is then associated with reduction in volume. The hypothetical relationship is illustrated in Fig. 1. Examples of this dissociation are to be seen in choriocarcinoma undergoing cytotoxic therapy. Tumours often continue to increase in size during the early stage of treatment. Later, when volume is diminishing, the halving times of the cell population (as indicated by the rate of hormone production) have been only 2–10 days when the respective volume halving times have been 30–60 days. Similarly, steady states of hormone excretion have been associated with tumours which continue to increase in volume. This suggests that tumour volume is only a poor guide to changes in the number of fertile cells in a tumour mass. Since clinical assessment of the response of tumours to therapy is largely based on observations of tumour volume, it is clear that it may be erroneous to conclude that a therapeutic agent has been ineffective against the proliferating cells of a tumour simply because tumour volume has failed to decrease.

*Action of anti-mitotic agents*

The action of anti-mitotic agents against proliferating cell populations can now be considered. For this it is convenient to define two hypothetical agents, both of which have the characteristic of killing cells as they prepare for mitosis during the period of drug exposure, but which have no effect on cells which do not opt to divide. They have ideal diffusion characteristics and reach the appropriate receptor sites in all tumour cells in effective concentration. Cells do not become resistant to their action either by adaptation or selection. They can be administered so that effective drug concentrations are attained immediately and sustained for the period of administration, but their effects cease immediately they are discontinued. " A " is highly selective and kills only dividing tumour cells whereas " B " is non-selective and does not discriminate between normal cells and tumour cells. Thus " A " is non-toxic and can be administered for indefinite periods, whereas " B " causes toxicity and can be allowed to act for only limited periods. " A " can be regarded as the ultimate in mitotic agents and has no real counterpart; " B " does not differ greatly from some existing agents and it may be compared with an anti-metabolite such as the folic acid antagonist methotrexate.

Methotrexate inhibits DNA synthesis, and cells which enter S phase, that is the phase of DNA replication (Howard and Pelc, 1953) during exposure to effective concentrations of this drug, fail to produce viable daughter cells (Bertino, 1963; Werkheiser, 1963, 1965). In its free form it is rapidly excreted and cells not engaged in DNA synthesis during its administration appear to suffer no loss of capacity to proliferate subsequently. For this reason, the fraction of cells in a population killed by methotrexate approximates to the fraction entering S phase during the period of treatment and the size of this fraction is therefore a function of the duration of treatment.

Since the duration of S phase is short in comparison with the period of action of hypothetical agent B in the present analysis, the number of cells which pass only a part of S phase in a period of drug exposure is small enough to be ignored.

*Fractional cell kill*

There is some evidence that the fraction of the total number of fertile cells which can be killed during a unit of time tends to remain constant for a particular population. Watson (1908) found that for a given concentration of disinfectant the time required to kill a bacterial population was a function of the initial size of the population. His observations have often been confirmed (Pittillo *et al.*, 1965; Hunt and Pittillo, 1967).

Studies of L 1210 murine leukaemia and of solid tumours (Skipper *et al.*, 1964; Wilcox *et al.*, 1965) of Hodgkins disease (Johnson and Brace, 1966) and of choriocarcinoma (Bagshawe, 1968) have been consistent, with the conception that the fraction of the tumour cell population which can be killed with antimitotic agents during an interval of time tends to remain constant over a wide range of population size.

If susceptibility to an agent is dependent on cell division it is clear that the potential rate at which a population can be destroyed (potential reduction rate) is directly related to the potential growth rate. The killing of dividing cells means that each mitosis results not in the addition of a cell but in the loss of a cell. If the fraction of surviving fertile cells which enter S phase in time $t$ is constant $(dN/dt) = k$ then the potential halving time is equal to the potential doubling time (Fig. 2).

FIG. 2.—Relationship between potential growth rate and potential kill rate for fast and slow growing tumours exposed to effective anti-mitotic agent. The reciprocal relationship holds because each mitosis results not in the addition, but in the loss of a cell during exposure to the agent.

*Time required to eliminate a population of known size and potential doubling time in the absence of spontaneous cell losses*

The time required to eliminate a numerically defined tumour cell population with hypothetical agent A can be calculated for various potential growth rates. The time required to eliminate a moderate sized tumour population of $10^{10}$ (approx. $2^{34}$) cells is indicated in Table II. The assumptions are that the rate of entry of cells into S phase is constant and unaltered by treatment and that no spontaneous loss of cells occurs. Even for rapidly proliferating tumours the duration of uninterrupted action required to eliminate the population is much longer than the total duration of chemotherapy usually given in clinical practice. For slowly proliferating tumours several years' continuous treatment would be necessary.

Antimitotic agents which block DNA synthesis can be given continuously in fully effective dosage for only about 4–5 days. To persist longer causes death; the organism as a whole is more vulnerable than its most susceptible tissues. Hypothetical agent B can therefore be proposed to kill all cells entering S phase of the cell cycle during, say, a 4 day period which then has to be followed by a rest phase-without treatment, to allow the recovery of normal tissues. Tumour growth is

TABLE II.—*Time Required to Eliminate a Tumour Cell Population of* $10^{10}$ (*approx.* $2^{34}$) *Assuming a Constant Daily Fractional Kill*

| (1) Tumour cell population halving time or doubling time (days) | (2) Fraction of tumour cell population eliminated each day | (3) Number of days required to reduce $10^{10}$ cells to $< 10^{0}$ |
|---|---|---|
| 0·5 | 0·75 | 17·5 |
| 1 | 0·5 | 35 |
| 2 | 0·25 | 70 |
| 5 | 0·1 | 175 |
| 10 | 0·05 | 350 |
| 20 | 0·025 | 700 |
| 50 | 0·01 | 1750 |
| 100 | 0·005 | 3500 |

resumed during the rest phase and the net effect of a complete treatment phase-rest phase cycle is determined by the respective durations of the two phases, by the loss of cells inflicted on normal and tumour populations, and by their respective rates of recovery.

FIG. 3.—Effect of alternating treatment and rest phases of equal duration on a proliferative cell population from which there are no spontaneous cell losses. The population size would remain constant.

If agent B were used in a therapeutic schedule in which the treatment and rest phases were of equal duration and if all the cells entering mitosis during the treatment phase were killed but those entering mitosis during the rest phase divided successfully, then the net effect of the whole cycle would be a steady state (Fig. 3). In practice, an intensive 4 day course of methotrexate has to be followed by a rest phase of about twice that duration. So that a population, exposed to a schedule of 4 days treatment followed by 8 days rest, would continue to grow, but at a slower rate than if untreated (Fig. 4). If the potential doubling time of the population were 4 days, then instead of increasing 8-fold in 12 days it would only increase 2-fold (Fig. 5). Thus an agent which is 100% efficient at killing mitosing cells during the period of its action would fail to arrest growth; such a situation in clinical practice would probably be interpreted as " drug resistance " since tumour volume would continue to increase.

Nor is there good reason to suppose that a combination of type B antimitotic agents used simultaneously would prove more effective than a single agent. If two agents act only on cells in S phase or in mitosis their simultaneous use can only effect an " overkill " of the same population. The combination of a type B antimitotic with, say, an alkylating agent, would perhaps increase the number of cells

FIG. 4.—Effect of alternating treatment and rest phases with respective durations in the ratio 1 : 2 on a proliferative cell population from which there are no spontaneous losses.

FIG. 5.—Effect of 4 day treatment and 8 day rest phases on a proliferative cell population with a potential doubling time of 4 days and no spontaneous losses. During the complete treatment-rest cycle the population increases 2-fold when in the absence of treatment it would increase 8-fold.

in the tumour population sustaining lethal damage, but it could be expected to exert a parallel effect on normal cell populations so that the rest phase would have to be extended, and it does not follow that a net gain would necessarily be achieved. There may, of course, be other valid reasons for using a combination of cytotoxic agents, such as an attempt to achieve full blockade of DNA synthesis where one agent alone is inadequate.

*Effect of spontaneous cell losses on effectiveness of anti-mitotic agents*

It is clear that if the model so far constructed were representative of all tumours, then type B antimitotic agents would be incapable of doing more than reducing the growth rate of any tumour, whereas in fact, agents such as methotrexate do eliminate some human tumours when used cyclically in schedules similar to that just described. If the foregoing analysis is correct this discrepancy can be attributed only to spontaneous cell losses and these must now be incorporated into the analysis. It seems reasonable to assume that spontaneous losses occur during both treatment and rest phases but it cannot be assumed that the rates of spontaneous loss are the same in the two phases. Since there is no firm basis for computing the rates separately, it is convenient to group all spontaneous losses into the rest phase and express them as a fraction of the total cells added by mitosis.

Fig. 6.—Effect of alternating treatment and rest phases with respective durations in the ratio 1 : 2 on a proliferative population from which (a) two thirds of the cells added by mitosis in the rest phase are lost spontaneously; (b) more than two thirds of the added cells are lost spontaneously.

When two-thirds of the cells added by mitosis during the rest phase are lost spontaneously and the duration of treatment and rest phases are in the ratio of 1 : 2 then the net effect of a complete treatment cycle with hypothetical agent B would be a steady state (Fig. 6a). If spontaneous cell losses exceed two thirds of cells added by mitosis in the rest phase then the population size would be reduced by the amount of that excess (Fig. 6b). If cell losses were less than two thirds of the added cells, the population would continue to show a net increase. Similar computations can be made for other treatment-rest phase ratios.

*Effect of anti-mitotic agents on normal proliferating tissues*

The duration of a course of antimitotic therapy is limited by the loss of alimentary tract mucosal cells and the reduction of circulating leucocytes and platelets. The patterns of cell proliferation in the intestinal crypts (Bizzozero, 1892; Friedman, 1945; Leblond and Stevens, 1948; Fry *et al.*, 1963) and the complexities of haemopoiesis (Osgood, 1954; Cronkite *et al.*, 1960; Lajtha *et al.*, 1962; Patt and Maloney, 1964) have been partially revealed. For the present purpose it is necessary to adopt an over simplified model which distinguishes only between (i) a precursor compartment which includes both fertile stem cells and the immediate precursors of the tissue functional cells and (ii) the fully differentiated, non-fertile, tissue functional cells.

By analogy with the analysis of the tumour population, cells are lost spontaneously from the precursor compartment into the differentiated compartment, and in a steady state, losses equal 100% of the cells added by mitosis. The spontaneous cell loss rate equals the potential growth rate and the potential kill rate with an antimitotic also equals the potential growth rate. Combined spontaneous and therapy induced losses from a precursor compartment therefore approach a limiting value equal to twice its potential growth rate.

This limiting rate would not be attained if therapy reduces the flux into the differentiated compartment. It is a common observation that the peripheral blood white cell and platelet counts do not usually fall until 3–4 days after starting treatment with full doses of an agent such as methotrexate, suggesting that normal losses from the differentiated compartment continue to be made good by replacements from the precursor compartment. It seems likely, therefore, that at least for a substantial part of a course of treatment, cell losses by differentiation from the precursor compartment are not greatly reduced and total losses may approach the limiting rate.

It cannot be assumed that the *potential* growth rate of a normal steady state population is its *maximum* growth rate. Cell production increases in response to various stimuli and a reduction in the number of cells in the differentiated cell compartment may be an appropriate direct or indirect stimulus to its respective stem cell precursors. If the number of cells preparing for mitosis increases during a course of anti-mitotic therapy, then the therapy induced kill rate also increases.

During the early part of the rest phase an increased rate of proliferation in normal precursor compartments aids rapid recovery from the losses incurred by anti-mitotic action. During or following the reconstitution of stem cell populations, cells become available for differentiation and finally, as the normal number of cells in the differentiated compartment is restored, so homeostatic mechanisms may be supposed to operate so that stem cell proliferation is reduced to the rate associated with the steady state.

Since some normal precursor compartments have higher potential kill rates and greater spontaneous losses than expanding tumour populations, the combined losses from these normal tissues are relatively greater than those from tumours during a treatment phase. But when a complete cycle, consisting of a treatment phase followed by a rest phase, does achieve a useful net effect (i.e. the fertile tumour cell population fails to recover its initial size by the time normal differentiated cell populations are fully restored), then it is clear that the advantage is gained in the rest phase (Fig. 7). The duration of the rest phase is critical since normal stem cell recovery is most rapid and outpaces the tumour cells in the early part of the rest phase, but is later depressed to its steady state level by the operation of homeostatic mechanisms. If the rest phase is unduly prolonged, the less is the likelihood of a useful net effect, because the tumour population continues to expand.

### DISCUSSION

The general applicability of analyses of the present type depends on the degree of dissociation between the conditions assumed and those which apply in reality.

The validity of the model of anti-mitotic action is clearly limited by the validity of the model of tumour growth and it is recognised that it is not possible to encompass processes of almost infinite complexity with a few highly simplified concepts.

It may also be argued that the validity of the present analysis is limited by the adoption of hypothetical, rather than real, anti-mitotic substances. But the present purpose is to attempt to define the potential value of agents whose action is confined to killing cells in, or preparing for, mitosis. Although hypothetical agent B, adopted for the analysis, corresponds to agents like methotrexate more closely than to alkylating agents, vinca alkaloids or irradiation, it is suggested that similar considerations probably apply to these agents also. Methotrexate is not

FIG. 7.—Predicted effects of anti-mitotic action on an expanding tumour cell population and on a normal steady state population (e.g. granulocytes and their precursors). The potential growth rates and therefore the potential reduction rates during therapy in the two populations are similar, but spontaneous losses are greater in the steady state population so that total losses during the treatment phase are also greater. If the two populations recover their initial size at the same time it is implicit that the steady state population has outpaced the expanding population during the early part of the rest phase before homeostatic mechanisms effectively limit its own growth.

| | |
|---|---|
| —·—·—·— | Tumour cell population. |
| – – – – – – | Steady state-precursor population. |
| ........... | Steady state-differentiated cell population. |
| PGR | Potential growth rate. |
| AGR | Actual growth rate. |
| SCLR | Spontaneous cell loss rate. |

notably more effective or less effective than other anti-mitotic agents, but its mode of action is such that the fraction of cells killed can be adequately defined on a temporal basis, whereas the effects of many other agents are temporally dispersed and less definable. Many notable studies of cell proliferation kinetics have been based on radiobiology and it is unfortunate that the effects of irradiation are, in this respect, less amenable to analysis than those of rapidly excreted anti-metabolites. It is also clear that real agents cannot be more effective than the hypothetical agents, except in so far as they possess a non-mitotic basis for a selective effect.

It might be argued that susceptible human tumours are sensitive because of certain intrinsic biochemical characteristics. If this were so, it might be anticipated that such tumours would be susceptible to drug regimen which have no marked effect on normal cell proliferation. However, this does not appear to occur. Nor is there clinical evidence of unusually prolonged drug action on susceptible tumours, as might be expected if there were selective retention of drug within their cells. Certain experimental tumours, it is true, have proved susceptible only to certain cytotoxic agents and this would suggest that the effective agents have an additional extra-mitotic action against these tumours; in either event this is not relevant to the general thesis of anti-mitotic action.

That tumours respond well at first to anti-mitotics but later continue to grow despite them is a frequent clinical sequence and it is usually attributed to the operation of adaptive and selective mechanisms. It is not to be denied that these mechanisms may indeed operate but the question is whether they usually account for the apparent failure which follows a promising start. The fact that the development of resistance to one agent is frequently accompanied by resistance to various other agents which act on different metabolic pathways (Hutchinson, 1963, 1965), seems to argue strongly against specific adaptive and selective mechanisms, but is consistent with the hypothesis that non-susceptibility to anti-mitotics is dependent on proliferation kinetics which are unfavourable to anti-mitotics in general.

The induction of resistance to methotrexate in bacterial and mammalian cells has been readily achieved *in vitro* by convincingly selective mechanisms, but it was only achieved with difficulty after several serial passages with the L 1210 mouse leukaemia cell line *in vivo* (Welch, 1959). Increased dihydrofolate reductase levels and impaired membrane transport of methotrexate found in mouse leukaemic cells have been implicated as mechanisms of acquired resistance, but their relevance to resistance by human tumour cells *in vivo* has not been established (Bertino, 1965; Werkheiser, 1965).

Bertino (1965) observed that the development of resistance by normal tissues to methotrexate had not been seen, and in this unit patients receiving intensive therapy for 2–3 years with methotrexate, 6-mercaptopurine, actinomycin-D and other agents, have shown no loss of sensitivity in their haemopoietic and mucosal responses to these agents. Moreover, in patients where intensive chemotherapy has failed to eliminate a choriocarcinoma, resistance by the tumour cell population has not been absolute; tumour growth rates have invariably been lower during chemotherapy than in rest periods. The tumours of relapsing patients frequently show undiminished sensitivity to repeated treatment with the same agent (Fig. 8). Choriocarcinoma, like Burkitt lymphoma, is sensitive to a wide range of cytotoxic agents, although it is true that it is more sensitive to two particular agents.

On the present analysis, the emergence of drug resistance would be anticipated on the basis of a reduced rate of spontaneous cell loss. There is no reason to assume that this rate remains constant during substantial changes in tumour volume. In large tumour masses the spontaneous cell death rate may be expected to be high, as recently demonstrated by Frindel *et al.* (1967), and thus a good initial effect might be anticipated. Later, as a tumour's volume diminishes, spontaneous losses may be so reduced that continued treatment does not result in a further net reduction of the number of fertile cells. With continued treatment it would be predicted that such a tumour would ultimately increase in volume but at a slower rate than it would have done in the absence of treatment. Alternatively, if treatment is discontinued after a tumour has shown an initial reduction in volume and is allowed to grow at its natural rate, back to its initial volume where spontaneous losses again exceed the critical value, then further treatment at this time would be expected to have an effect comparable to, but not necessarily as great as, that achieved initially. Thus, where the spontaneous losses are readily depressed below the critical value, drug resistance appears to occur, and the difference achieved by frequently repeated courses of chemotherapy and by less frequently repeated courses may be marginal. By contrast, where spontaneous losses remain above the critical value, then further treatment can result in a further net reduction in the fertile tumour cells, but only provided the interval between successive

FIG. 8.—Human chorionic gonadotrophin (HCG) excretion by a patient with choriocarcinoma (Case No. 56). The patient relapsed following each of three series of treatments but has remained in remission following a longer fourth series of treatments with the same anti-mitotic agents as used earlier. The rate of tumour destruction, as judged by the rate of fall of HCG excretion, was similar with each series of treatments suggesting that failure to eliminate the tumour with the earlier courses of treatment was not the result of adaptive or selective mechanisms.

courses is kept to a minimum. Differences between palliative and potentially curative chemotherapeutic regimen may therefore have some basis.

It would not be justified, however, to conclude that spontaneous cell losses are always relatively greater the larger the tumour mass. These losses result from a variety of mechanisms and the effect of treatment and change of volume on each route of cell loss may differ substantially. Host response factors would be expected to be most effective in causing tumour cell losses when the ratio of tumour-host interface area to tumour volume is maximal, that is, when tumour volume is minimal.

Except for tumours with a sustained high level of spontaneous cell death, the present analysis provides no ground for supposing that cancer can be effectively controlled with systemic anti-mitotic agents, however efficient, if used alone. The general toxic effects of anti-mitotics are often too high a price to pay for a slight retardation of growth. But this is not to say they have no place. It is now widely recognised that factors which differentially stimulate the entry of normal or malignant cells into mitosis, or differentially retard them, are potentially valuable.

It is also necessary to consider the possible contribution of immune mechanisms to spontaneous cell losses and if possible to ensure that losses by this route are augmented and not diminished by therapy. There is a *prima facie* case for supposing that anti-mitotic agents might reduce the effectiveness of an immune reaction to a tumour, yet there is some evidence from choriocarcinoma that the contribution from immune mechanisms is not necessarily obliterated by chemotherapy. Preliminary evidence suggests that a marked mononuclear reaction to gestational choriocarcinoma appears to be associated with a better prognosis in chemotherapy-treated patients than a poor reaction (Elston, 1968, unpublished data). It is therefore interesting that the agents used successfully in the treatment of this tumour, methotrexate and actinomycin-D, have been observed to facilitate lymphocyte interaction with target cells (Svet-Moldavsky *et al.*, 1968).

The margin between success and failure in the chemotherapy of gestational choriocarcinoma is often narrow and precarious, although in a majority of patients it is fortunately possible to eliminate this tumour (Bagshawe, 1967; Hertz, 1967). Despite the rather regular failure of anti-mitotic agents to eliminate most other tumours, it would seem wrong to assume that the margin of failure is necessarily a wide one. The value of anti-mitotic action on many tumours may not be realizable until additional means of killing tumour cells become available.

### SUMMARY

Changes in normal and malignant cell populations as a consequence of spontaneous and therapeutically induced cell losses have been analysed using a hypothetical, fully effective, anti-mitotic agent, with a temporally defined period of action, and compared with the effects of methotrexate on gestational choriocarcinoma.

Changes in the number of fertile tumour cells are not necessarily reflected by changes of tumour volume.

The limiting rate at which an anti-mitotic agent can reduce a cell population is equal to its potential growth rate. The potential reduction rates of certain normal tissues are higher than those of most tumours.

In the absence of spontaneous cell losses, an agent which is specifically lethal to dividing tumour cells would need several years of continuous administration to eliminate a moderate sized human tumour with a low potential growth rate; a non-specific anti-mitotic agent could reduce, but not abolish, the rate of increase of a tumour population, when used in maximum tolerated dosage.

The rates of spontaneous cell loss in normal proliferating steady state tissues equal their respective potential growth rates and exceed those in expanding tumour populations. Spontaneous losses in tumours occur by various routes and probably change with size and other factors, within the same tumour.

When the spontaneous cell loss rate exceeds a value which is critical for each therapeutic regimen, then anti-mitotic therapy can reduce tumour population size. In those tumours which are eliminated by chemotherapy there is evidence of high rates of spontaneous cell loss. The higher the potential growth rate and the lower the actual growth rate, the better the opportunity for anti-mitotic agents.

Total cell losses from certain normal proliferating tissues during anti-mitotic therapy are relatively greater than from most tumours but, where therapy achieves a useful net effect, normal tissue recovery outpaces tumour recovery until normal tissue populations have been reconstituted.

I wish to thank Charing Cross Hospital Research Sub-committee for its support and Dr. G. G. Steel, Dr. M. C. Pike, and Dr. B. M. Liversage for helpful discussions on topics related to this paper.

### REFERENCES

BAGSHAWE, K. D.—(1967) in ' Choriocarcinoma '. Transactions of a Conference of the International Union against Cancer. Edited by J. F. Holland and M. M. Hreshchyshyn. Berlin (Springer Verlag).—(1968) Transcript of the Proceedings of the Fourth Rochester Trophoblast Conference. In the Press.

BASERGA, R.—(1965) *Cancer Res.*, **25**, 581.

BASERGA, R., HENEGAR, G. C., KISIELESKI, W. E. AND LISCO, H.—(1962) *Lab. Invest.*, **11**, 360.

BERTINO, J. R.—(1963) *Cancer Res.*, **23**, 1286.—(1965) *Cancer Res.*, **25**, 1614.

BIZZOZERO, G.—(1892) *Arch. mikrosk. Anat. EntwMech.*, **40**, 325.

BREUR, K.—(1966) *Eur. J. Cancer*, **2**, 157.

BURTON, A. C.—(1966) *Growth*, **30**, 157.

COLE, J. W. AND MCKALEN, A.—(1961) *Gastroenterology*, **41**, 122.

COLLINS, V. P.—(1962) *Cancer, N.Y.*, **15**, 387.

COLLINS, V. P., LOEFFLER, R. K. AND TIVEY, H.—(1956) *Am. J. Roentg.*, **76**, 988.

COOPER, E. H., FRANK, G. L. AND WRIGHT, D. H.—(1966) *Eur. J. Cancer*, **2**, 377.

CRONKITE, E. P., BOND, V. P., FLIEDNER, T. M. AND KILLMANN, S. A.—(1960) in ' Haemopoiesis '. Edited by G. E. W. Wolstenholme and M. O'Connor. London (Churchill), p. 70.

FRIEDMAN, N. B.'(1945) *J. exp. Med.*, **81**, 553.

FRINDEL, E., MALAISE, E. P., ALPEN, E. AND TUBIANA, M.—(1967) *Cancer Res.*, **27**, 1122.

FRY, R. J. M., LESHER, S., KISIELESKI, W. E. AND SACHER, G. (1963) in ' Cell Proliferation ', A Guiness Symposium. Edited by L. F. Lamerton and R. J. M. Fry. Oxford (Blackwell).

GARLAND, L. H., COULSON, W. AND WOLLIN, E.—(1963) *Cancer, N.Y.*, **16**, 694.

HADDOW, A.—(1938) *J. Path. Bact.*, **47**, 553.—(1963) *Proc. R. Soc. Med.*, **56**, 629.

HERTZ, R.—(1967) in ' Choriocarcinoma '. Transactions of a Conference of the International Union against Cancer. Edited by J. F. Holland and M. M. Hreshchyshyn. Berlin (Springer Verlag).

HOWARD, A. AND PELC, S. R.—(1953) *Heredity, Lond.*, Suppl., **6**, 261.

HUNT, D. E. AND PITTILLO, R. F.—(1967) *Appl. Microbiol.*, **15**, 334.

HUTCHISON, D. J.—(1963) in ' Advances in Cancer Research '. Edited by A. Haddow and S. Weinhouse. London (Academic Press), Vol. 7.—(1965) *Cancer Res.*, **25**, 1581.

IVERSEN, O. H.—(1967) *Eur. J. Cancer*, **3**, 389.

JOHNSON, H. A., RUBINI, J. R., CRONKITE, E. P. AND BOND, V. P.—(1960) *Lab. Invest.*, **9**, 460.

JOHNSON, R. E. AND BRACE, K. C.—(1966) *Cancer, N.Y.*, **19**, 368.

KILLMANN, S. A., CRONKITE, E. P., ROBERTSON, J. S., FLIEDNER, T. M. AND BOND, V. P.—(1963) *Lab. Invest.*, **12**, 671.

KLEIN, G.—(1961) in ' Biological Approaches to Cancer Chemotherapy '. Edited by R. J. C. Harris. London (Academic Press).

LAJTHA, L. G., OLIVER, R. AND GURNEY, C. W.—(1962) *Br. J. Haemat.* **8**, 442.
LAW, L. W.—(1956) *Cancer Res.*, **16**, 698.
LEBLOND, C. P. AND STEVENS, C. E.—(1948) *Anat. Rec.*, **100**, 357.
MAUER, A. M.—(1964) *Lancet*, ii, 675.
MAYNEORD, W. V.—(1932) *Am. J. Cancer*, **16**, 841.
MENDELSOHN, M. L.—(1963) in ' Cell Proliferation, A Guiness Symposium '. Edited by L. F. Lamerton and R. J. M. Fry. Oxford (Blackwell Scientific Publishers), p. 190.
OSGOOD, E. E.—(1954) *Blood*, **9**, 1141.
PATT, H. M. AND BLACKFORD, M. E.—(1954) *Cancer Res.*, **14**, 391.
PATT, H. M., MALONEY, M. A.—(1964) *Ann. N.Y. Acad. Sci.*, **113**, 513.—(1959) in ' The Kinetics of Cellular Proliferation '. Edited by F. Stohlman. New York (Grune and Stratton), p. 201.
PITTILLO, R. F., SCHABEL, F. M., WILCOX, W. S. AND SKIPPER, H. E.—(1965) *Cancer Chemother. Rep.*, **47**, 1.
POTTER, M.—(1958) in ' Screening Procedures for Experimental Chemotherapy '. Edited by C. C. Stock. *Ann. N.Y. Acad. Sci.*, **76**, 630.
SHREK, R.—(1936) *Am. J. Cancer*, **28**, 345.
SCHWARTZ, M.—(1961) *Cancer, N.Y.*, **14**, 1272.
SKIPPER, H. E., SCHABEL, F. M. AND WILCOX, W. S.—(1964) *Cancer Chemother. Rep.*, **35**, 1.
SPRATT, J. S. AND SPRATT, T. L.—(1964) *Ann. Surg.*, **159**, 161.
STEEL, G. G.—(1967) *Eur. J. Cancer*, **3**, 381.
SVET-MOLDAVSKY, G. J., KADAGHIDZE, Z. G., SURA, S. N.—(1968) *Lancet*, i, 148.
TITUS, J. L. AND SHORTER, R. G.—(1965) *Archs Path.*, **79**, 324.
WATSON, H. E.—(1908) *J. Hyg., Camb.*, **8**, 536.
WELCH, A. D.—(1959) *Cancer Res.*, **19**, 359.—(1965) *Ann. N.Y. Acad. Sci.*, **123**, 19.
WELIN, S., YONKER, J. AND SPRATT, J. S.—(1963) *Am. J. Roentg.*, **90**, 673.
WERKHEISER, W. C.—(1963) *Cancer Res.*, **23**, 1277.—(1965) *Cancer Res.*, **25**, 1608.
WILCOX, W. S., GRISWOLD, D. P., LASTER, W. R., SCHABEL, F. M. AND SKIPPER, H. E.—(1965) *Cancer Chemother. Rep.*, **47**, 27.